# Asymmetric Sensory Reweighting in Human Upright Stance

**DOI:** 10.1371/journal.pone.0100418

**Published:** 2014-06-24

**Authors:** David Logan, Tim Kiemel, John J. Jeka

**Affiliations:** 1 Department of Kinesiology, University of Maryland, College Park, Maryland, United States of America; 2 Neuroscience and Cognitive Science Program, University of Maryland, College Park, Maryland, United States of America; 3 Biomedical Engineering Graduate Program, University of Maryland, College Park, Maryland, United States of America; 4 Department of Kinesiology, Temple University, Philadelphia, Pennsylvania, United States of America; VU University Amsterdam, Netherlands

## Abstract

To investigate sensory reweighting as a fundamental property of sensor fusion during standing, we probed postural control with simultaneous rotations of the visual scene and surface of support. Nineteen subjects were presented with pseudo-random pitch rotations of visual scene and platform at the ankle to test for amplitude dependencies in the following conditions: low amplitude vision: high amplitude platform, low amplitude vision: low amplitude platform, and high amplitude vision: low amplitude platform. Gain and phase of frequency response functions (FRFs) to each stimulus were computed for two body sway angles and a single weighted EMG signal recorded from seven muscles. When platform stimulus amplitude was increased while visual stimulus amplitude remained constant, gain to vision increased, providing strong evidence for inter-modal reweighting between vision and somatosensation during standing. Intra-modal reweighting of vision was also observed as gains to vision decreased as visual stimulus amplitude increased. Such intra-modal and inter-modal amplitude dependent changes in gain were also observed in muscular activity. Gains of leg segment angle and muscular activity relative to the platform, on the other hand, showed only intra-modal reweighting. That is, changing platform motion amplitude altered the responses to both visual and support surface motion whereas changing visual scene motion amplitude did not significantly affect responses to support surface motion, indicating that the sensory integration scheme between somatosensation (at the support surface) and vision is asymmetric.

## Introduction

The fusion of multiple sensory inputs for control of human upright stance has been studied extensively over the last 30 years (for reviews, see [1]–[3]). It is now a generally held view that visual, vestibular and somatosensory inputs are dynamically re-weighted to maintain upright stance as environmental or nervous system conditions change (e.g., sensory deficits) [4]–[6]. Environmental changes such as moving from a light to a dark environment or from a fixed to a moving support surface (e.g., onto a moving walkway at the airport) require an updating of sensory weights to current conditions so that muscular commands are based on the most precise and reliable sensory information available [2], [7]–[8].

Early studies of sensory reweighting focused on removal or attenuation of a sensory input by closing the eyes or techniques such as sway-referencing the support surface (e.g., [9]), with the implicit goal of determining how the nervous system adapted to a neurological deficit such as bilateral vestibular loss. Efforts have also focused on properties of sensory reweighting in healthy individuals, for example, by perturbing a sensory input at a particular frequency of motion (e.g., [5]–[6]). As the amplitude of the sensory perturbation increases, postural sway amplitude does not match the increase, indicated by a decrease in gain (sway amplitude/perturbation amplitude). The interpretation of a change in gain is that as a perturbation of a sensory input increases in amplitude, the sensory input becomes a less reliable indicator of self-motion and the postural control system must downweight its influence (i.e., reduce gain) to remain upright. Without downweighting, a perturbation of increasing amplitude would eventually lead to loss of upright equilibrium. In the same vein, the nervous system upweights a sensory input when the perturbation of that input decreases in amplitude because the sensory input becomes a more reliable indicator of self-motion.

Sensory reweighting has also shed light on how the nervous system fuses multiple sensory inputs simultaneously [5]–[6], [10]. By presenting simultaneous sensory perturbations of touch and vision of varying amplitudes, an intra-modal and inter-modal dependency was revealed [6]. By increasing amplitude of a visual driving signal while keeping light touch stimulus amplitude constant (and vice-versa), gains of postural sway relative to vision (and touch) dropped, indicating intra-modal reweighting. Additionally, inter-modal reweighting was observed when gains to vision increased, despite a constant visual amplitude, while increasing the amplitude of touch signal only (and vice versa). We refer to this effect of stimulus amplitude on gain as inverse gain reweighting. Similar interactions have been observed between support-surface and vestibular sensory inputs [11].

In the current study, we use a visual perturbation (rotation of a virtual visual scene) and a platform perturbation (rotation of the support surface) to understand the inter-modal relationship between the weighting of vision and somatosensation (e.g., ankle proprioception, foot tactile sensation) for the control of upright stance. Movement of the visual scene is a purely sensory perturbation. Visual-scene movement changes the relative motion between the person and the visual scene, which changes visual inputs to the nervous system, which then changes the nervous system’s activation of muscles, resulting in changes in sway kinematics. A platform perturbation also acts as a sensory perturbation. Movement of the platform changes the relative motion between the person and the platform, which changes, for example, ankle proprioceptive inputs to the nervous system, which then changes the nervous system’s activation of muscles, again resulting in changes in sway kinematics. However, a platform perturbation is also a mechanical perturbation. Due to intrinsic (passive) musculotendon stiffness and damping at the ankle, a platform perturbation has a direct effect on sway kinematics that is not mediated by changes in muscle activation. The purely sensory nature of a visual-scene perturbation and the dual sensory/mechanical nature of a platform perturbation are described in the posture model of Peterka [5]. Based on responses to visual-scene and platform perturbations, Peterka concluded that the mechanical component of a platform perturbation is relatively small. Our hypotheses below are based on the platform perturbation being primarily sensory in nature. However, we will be attentive to the possibility that any deviations between our results and these hypotheses may be due to the platform perturbation’s mechanical component (see Discussion).

Sinusoidal pitch rotations of the visual scene have shown that visually induced sway amplitudes saturate as visual scene amplitude is increased, and this visually induced sway was approximately four times larger when the support surface was sway-referenced compared to fixed [12]. Additionally, gain to an anterior/posterior (A/P) visual stimulus has been shown to increase slightly when lateral platform perturbations are applied [10]. Increasing the amplitude of pseudorandom pitch platform rotations, however, cause robust decreases of platform-induced postural sway in a range of visual conditions such as eyes closed or sway-referencing of the visual scene [5]. The need exists, however, to study the use of both somatosensation (at the support surface) and vision during simultaneous, uncorrelated motion of the platform and visual scene.

Here we investigate the interaction of vision and the somatosensory system at the surface of support and predict that this interaction is governed by the same mechanism previously seen with other modalities [5], [11]. Our primary measures of interest are the gains of EMG and kinematic responses to visual-scene and platform perturbations. Based on a previous study with a visual perturbation [13], we first hypothesize that EMG responses across different muscles are coordinated such that these responses can be characterized by a single weighted EMG signal (see Methods). We describe sway kinematics using leg and trunk angles in the sagittal plane. Changes in EMG or kinematic gains across conditions with different perturbation amplitudes indicate nonlinearity in the postural control system. We hypothesize that this nonlinearity primarily reflects sensory reweighting so that: 1) decreases in gain to both visual and support surface perturbations are observed when the perturbation of the given sensory modality increases (intra-modal reweighting), 2) increases in gains are observed to each sensory perturbation when the perturbation of the different sensory modality increases (inter-modal reweighting) and 3) percentage gain changes across conditions are the same for EMG, leg-angle and trunk-angle responses. Properties 1 and 2 correspond to inverse gain reweighting, which is the primary signature of reweighting addressed in the literature (e.g., [5]–[6]). Property 3 follows from the joint input-output method of identifying different portions of the postural control feedback loop [13]–[16]. The key idea is that the relationship between EMG and kinematic responses to a sensory perturbation reflect the properties of how muscle activation produces movement (the *plant* in terms of control theory), not the properties of neural feedback such as sensory integration (see Eq. 3 and the accompanying text in [16] for the reasoning behind this idea). Therefore, if changes in gain across conditions are due to sensory reweighting and not nonlinearities in the plant, then the relationship between EMG and kinematic responses will not change across conditions. Under our assumption of a single weighted EMG signal, this implies that EMG, leg and trunk gains will change by the same percentage.

## Materials and Methods

### Ethics Statement

This study followed the principles of the Declaration of Helsinki for human subject protection. The protocol and consent form were approved by the Internal Review Board at the University of Maryland, College Park. Written informed consent was obtained from each participant.

### Subjects

Nineteen healthy University of Maryland Kinesiology students including ten males and nine females were used in this study. Subjects ranged in age from 18 to 30 years with a mean age of 21.3±3.3 years at the time of the study. All subjects were self reported to have no history of balance disorders, and were not using prescription drugs that affect balance. Additionally, subjects had no history of surgical procedures involving the feet, ankles, knees, hips, back, brain, spinal cord or inner ear.

### Apparatus

#### Virtual reality environment

**Figure 1:**
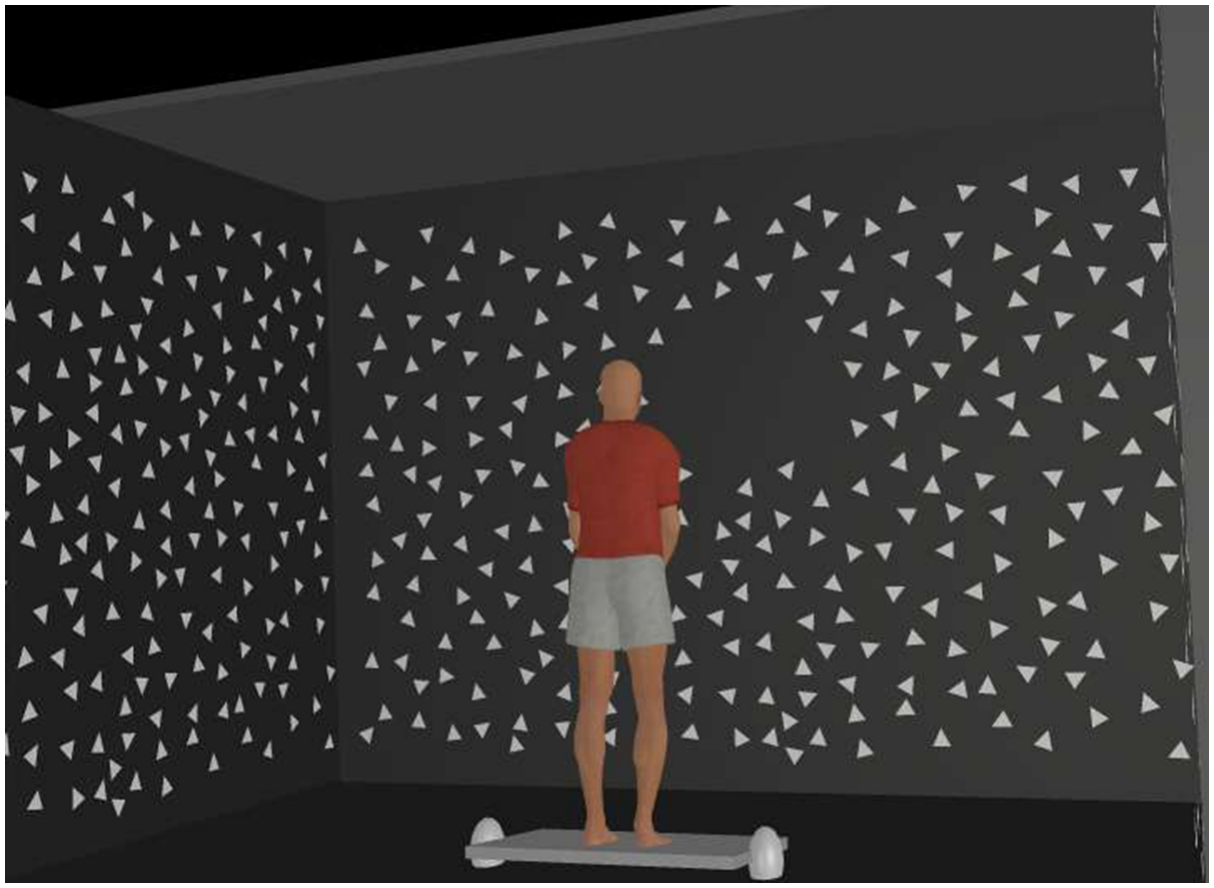
Visual cave and platform. The three-panel virtual visual scene consisting of randomly oriented triangles underwent a pitch rotation about an axis through the subjects’ ankles.

As seen in Figure 1, a visual cave simulating an enclosed environment consisting of a three-wall, virtual display with visual-stationary roof and floor was used. The walls of the cave were translucent screens whose display dimensions for all three walls were 244 cm wide by 305 cm high (Fakespace, Inc, USA). Subjects stood facing the front wall from a distance of 107 cm, and the side displays were located approximately 1 m from the subjects left and right. The roof of the cave consisted of a black, metallic surface that was 230 cm wide and extended approximately 10 cm past the subject’s head to create an enclosed environment. The dark green floor of the cave spanned the cave’s interior with an embedded force platform (model 4060-08, Bertec, Inc, USA).

The visual display was created using CaveLib software (Mechdyne, Inc, USA) with projection by JVC projectors (Victor Company, Japan) onto mirrors that reflected and rear-projected onto the three translucent screens. The visual display consisted of 500 randomly-distributed white triangles (1.52×1.52×2.16 cm) per wall on a black background, updated at 60 Hz. No triangles were placed within a 30 cm-radius circle whose center was directly in front of the subject’s eyes. This circle was created to suppress aliasing effects in foveal vision [17]. The visual display rotated about the axis through the subject’s ankle joints. A positive/negative signal corresponded to an anterior/posterior rotation.

#### Rotating platform

Subjects stood on a platform that rotated in the A–P direction about an axis that was coaxial with the subjects’ ankle. Platform motion was computer controlled by a digital servo-motor system (Compumotor Gemini GV6K, Parker Hannifin, Inc, USA). Across conditions and frequencies, average recorded platform gain from stimulus input was.73 while phase was −56° (*see gain and phase below*) meaning that actual platform motion was smaller and delayed from programmed platform motion. For this reason, recordings of actual platform motion were used to compute FRFs of the variable of interest from the platform stimulus.

#### Kinematics

Infrared emitting diodes were placed on the right ankle (lateral malleolus), right knee (fibular head), right hip (greater trochanter) and the right shoulder (acromion process) and measured at 120 Hz by a 3-camera Optotrak system (Northern Digital, Inc.) placed approximately 4 meters behind the subject. Platform angle was recorded midway between medial/lateral extremes of the platform and in line with the axis of rotation of the platform. These data were stored on a personal computerfor offline analysis.

#### EMG

EMG recordings of seven muscles were taken from the belly of the following muscles on the right side: soleus, gastrocnemius, tibialis anterior, biceps femoris, rectus femoris, lumbar erector spinae, and lower rectus abdominus. Electrodes were placed in accordance with sites proposed by SENIAM. Skin preparation included shaving, light abrasion with sandpaper and cleaning with an alcohol wipe. Pre-gelled, circular Al/AgCl electrodes with a 154 mm^2^ conducting area were used with an inter-electrode distance of 2 cm (Blue Sensors type M, Ambu, Denmark).

The first nine subjects were collected using Telemyo (Noraxon, USA) unit with an analog feed from its receiver unit into an Optotrak Data Acquisition Unit (ODAU) sampled at 1020 Hz. The Telemyo has an internal band-pass filter from 16–500 Hz. The last ten subjects were collected using a Zerowire (Aurion, IT) wireless unit with an internal band-pass filter from 10–1000 Hz sampled at 1020 Hz by an analog feed into an ODAU unit. There were not differences between the first nine subjects, last ten subjects, and all nineteen subjects in the signal processing outcomes described below.

### Procedures

Subjects were instructed to stand upright on a movable platform in the virtual reality cave with feet placed equidistant from the body’s sagittal midline. Distance between feet was approximately 11% of the subject’s height to allow 14° of external rotation at each foot [18]. The inside of the ankles were placed at the A–P rotational axis of the platform and arms were crossed across the front of the waist with hands comfortably clasped (Fig. 1). Prior to the beginning of each trial, subjects were instructed to stand comfortably without rigidity and maintain gaze within the blank circle on the front wall. Subjects were informed the trial was to begin and the simultaneous perturbations were started via external trigger with variable delay to avoid start-up effects.

Stimuli were created offline using Matlab (Mathworks, Inc, USA) and executed simultaneously with a custom LabView program (National Instruments, USA). The low amplitude platform signal was a 4 cycle, 1° peak to peak Pseudo Random Ternary Sequence (PRTS) [5], [19] that was shifted so that approximately 75% of the signal was above 0 (i.e., more of the signal was in the positive/anterior direction). To make the platform and visual stimuli uncorrelated, the second and fourth cycles of the low amplitude visual stimulus were multiplied by −1. A lag was then added to the signal by removing the first quarter of each cycle and shifting it to the end of its respective cycle. The visual stimulus was then re-scaled so that its peak to peak value was 1°. The high amplitude (4° peak to peak) visual and platform stimuli were created in the same manner.

The experimental design consisted of three conditions of visual and platform rotation at low or high amplitudes randomized within four blocks. The four trials for each condition lasted 242 seconds each, and a two minute period of seated rest was required between trials. The conditions were: 1) low vision-high platform, 2) low vision-low platform, and 3) high vision-low platform and referred to as 1v∶4p, 1v1∶1p and 4v∶1p, respectively, in figures and remaining text.

### Signal Processing

#### FRFs of segment angles

Leg segment angle was the angle formed from vertical by A–P hip displacement relative to A–P ankle displacement. Trunk segment angle was calculated by measuring the angle from vertical created by the A–P displacement of the shoulder relative to the A–P displacement of the hip.

To capture responses of the segments from visual and support surface stimuli separately, Fourier transforms of the detrended (mean removed) stimulus angles (*x(t)*) and detrended segment A–P angles (*y(t)*) were calculated. One-sided power spectral densities (PSDs) and cross spectral densities (CSDs) were calculated using a single 242 second rectangular window in a discrete Fourier transform between each segment angle with each of the two stimulus signals. PSDs and CSDs were then averaged across trials in each condition for each subject. PSDs and CSDs at nonzero stimulus frequencies were extracted and binned for each subject. Bin averages were based on binning the platform stimulus frequencies up to 3.984 Hz (frequency prior to 4 Hz) on a logarithmic scale [16]. Nonzero stimulus frequencies of visual stimuli were odd multiples of 1/121 Hz (at 1/121 Hz, 3/121 Hz …) and nonzero stimulus frequencies of platform stimuli were odd multiples of 1/60.5 Hz (at 1/60.5 Hz, 3/60.5 Hz …). Stimulus frequencies in the following ranges created the eight bins for platform stimuli:.017,.050−.083,.116−.215,.248−.380,.410−.711,.744 to 1.273, 1.306–2.198, and 2.231–3.984 Hz.. Bin ranges were created to make identical bin averages for both vision and platform stimuli of.017,.066,.165,.314,.562, 1.008, 1.752, and 2.991 Hz. To do so, stimulus frequencies in the following ranges created the eight bins for visual stimuli:.0083−.0248,.0413−.0909,.1074−.2231,.2397−.3884,.4050−.7190,.7355–1.2810, 1.2975–2.2066 and 2.2231–3.7603 Hz. We use average frequency in each bin to plot gain and phase as a function of frequency.

Using these binned PSDs and CSDs, complex coherence was calculated as 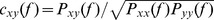. Frequency response functions (FRFs) of leg and trunk segment angles from both the visual and platform signal stimuli were calculated yielding four FRFs for each condition. The FRF was defined as 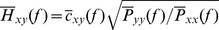
where 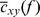 is the across-subjects mean complex coherence while 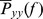 and 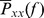 are across-subjects geometric mean PSDs [16].

### FRFs of Weighted EMG

In addition to responses of kinematic variables we also sought to relate changes in visual and platform stimuli to changes in muscle activations. To best investigate changes in muscle activity during simultaneous changes in both visual and platform stimuli, we obtained (through optimization) those weights of individual muscles of each subject that maximized the linear relationship (coherence) between the stimuli and a weighted muscle signal. FRFs of the weighted muscle signal to the visual and platform stimuli were then computed to characterize the relationship between stimuli and changes in muscle activity. These weighting methods have been used previously in investigations focused on identifying portions of the postural control feedback loop [13], [16], and we use them here for the first time in an investigation of sensory reweighting.

As it was initially unclear whether responses of those muscles primarily acting at the ankle, hip or both ankle and hip would best relate to changes in visual and platform stimuli, the weighted EMG signals to ankle muscles, hip muscles and all muscles were computed. For brevity, we first describe an EMG signal (*weighted ankle EMG*) composed of only ankle muscle EMGs and then note the muscles used in the other weighting schemes.

A *weighted ankle* EMG signal using the ankle muscles (soleus, gastrocnemius and tibialis anterior) was created by utilizing the Matlab optimization toolbox function FMINCON (inferior-point algorithm) with multiple sets of initial weights. EMG signals were first detrended, rectified and normalized by dividing by their root mean square values computed from all trials for the given subject. Due to the ensuing signal processing, this normalization did not affect the final results. Weights *w_j_* in the following equation were used to maximize the averaged coherence of each perturbation signal *v(t)* and the weighted ankle EMG signal *u(t)* =  w_1_u_1_(t) + w_2_u_2_(t) + w_3_u_3_(t) with EMG signals *u*1(*t*), *u*2(*t*), and *u*3(*t*) from the three ankle muscles. Averaging complex coherence (*c_vu_)* across all conditions and then averaging (magnitude squared) coherence *|c_vu_|^2^* across the eight frequency bins allowed calculation of average coherence of the muscle signal to each perturbation. Coherence maxima of the two perturbations were then averaged together to allow a single signal (*v(t)*) for FMINCON to optimally weigh the three ankle muscles. Additionally, these muscle weightings were constrained so that posterior muscle *w_j_*≥0, anterior muscle *w_j_*≤0 and |*w_1_*|+|*w_2_*|+|*w_3_*| = 1. Finally, each subject’s weighted ankle signal *u*(t) was normalized by mean response amplitude across conditions and amplitude of stimuli dictated by power at stimulus frequencies (Kiemel et al.2008). The same method was used to calculate the *weighted hip* EMG using signals obtained from biceps femoris, rectus femoris, rectus abdominus and erector spinae muscles while the *weighted all-muscle* EMG signal was calculated using EMG from all seven muscles.

FRFs of these weighted EMG signals to the stimulus signals were calculated using the same Fourier methods as FRFs of segment angles. In these FRFs, the input x(t) was perturbation signal and the output y(t) was the weighted EMG signal to yield two FRFs for each of the three weighted EMG signals (ankle, hip or all-muscle*).* Additionally, (magnitude squared) coherence was computed from 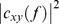 for these weighted muscle signals for an indication of the linear relationship between perturbation×and muscle signal y. Adjusted for a third signal z, the partial coherence between×and y is 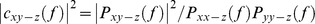 where 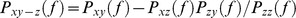 is the conditional spectral density [20]. Partial coherence reveals the strength of the linear relationship between signals×and *y* that is not due to their linear relationships with *z*.

### Robust Muscle Weighting Method

To confirm that our results were not dependent on the method of weighting EMG; we calculated FRFs of these weighted EMG signals with different weighting schemes. In addition to our primary method, we weighted EMG to the averaged maximum coherence without constraining the signs of the weights and through an equal magnitude weighting that required weights of posterior muscles to be positive and anterior muscles to be negative. With alpha = .05 and the same bootstrapping method used for gain and phase comparisons (see *Statistics* below), 1124/1152 (98%) comparisons between constrained, unconstrained and equal parts weighting were not significantly different. To further test the robustness of this weighting method; comparisons were made of results found when these weights maximized coherence to visual drive alone, platform drive alone, and average of both stimuli. The comparisons yielded 3036/3456 (88%) non-significant differences between gain and phase calculated using these three methods. As a result, the weighted all-muscle EMG signal whose coherence is maximized to the average of both stimuli is used in computing the FRFs presented.

### Statistics: Gain and Phase

The outcome measures used to characterize these FRFs were gain and phase. Gain is the absolute value of 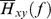 and phase is the argument of 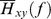, converted to degrees. Gain greater than 1 indicates amplitude of *y(t)* was greater than amplitude of *x(t)* while a positive phase indicates that *y*(*t*) was phase advanced relative to *x*(*t*) at that particular frequency. Gain and phase of FRFs are plotted with error bars representing ± standard deviation of 10,000 bootstrapped resamplings using the percentile*-t* method [21].

Bootstrapped 95% confidence intervals of log gain and phase were calculated using the percentile-t method with 4000 bootstrap resamples and 400 nested bootstrap resamples for variance estimation [21]–[22]. To investigate reweighting relationships at individual frequencies, pair-wise gain ratios and phase differences were bootstrapped at each frequency bin. If these bootstrapped 95% confidence intervals of gain included one, the gain comparison was deemed not statistically different at α = .05 (p<.05). Likewise, bootstrapped 95% confidence intervals of phase differences were deemed not significantly different at α = .05 if 0° was included in the confidence interval. To test for main condition effects for each output variable (leg segment, trunk segment and EMG) and interactions between condition and output variable, log-gain and phase were averaged across the eight frequency bins and tested using the bootstrap method of [23] at significance level α = .05.

## Results

**Figure 2:**
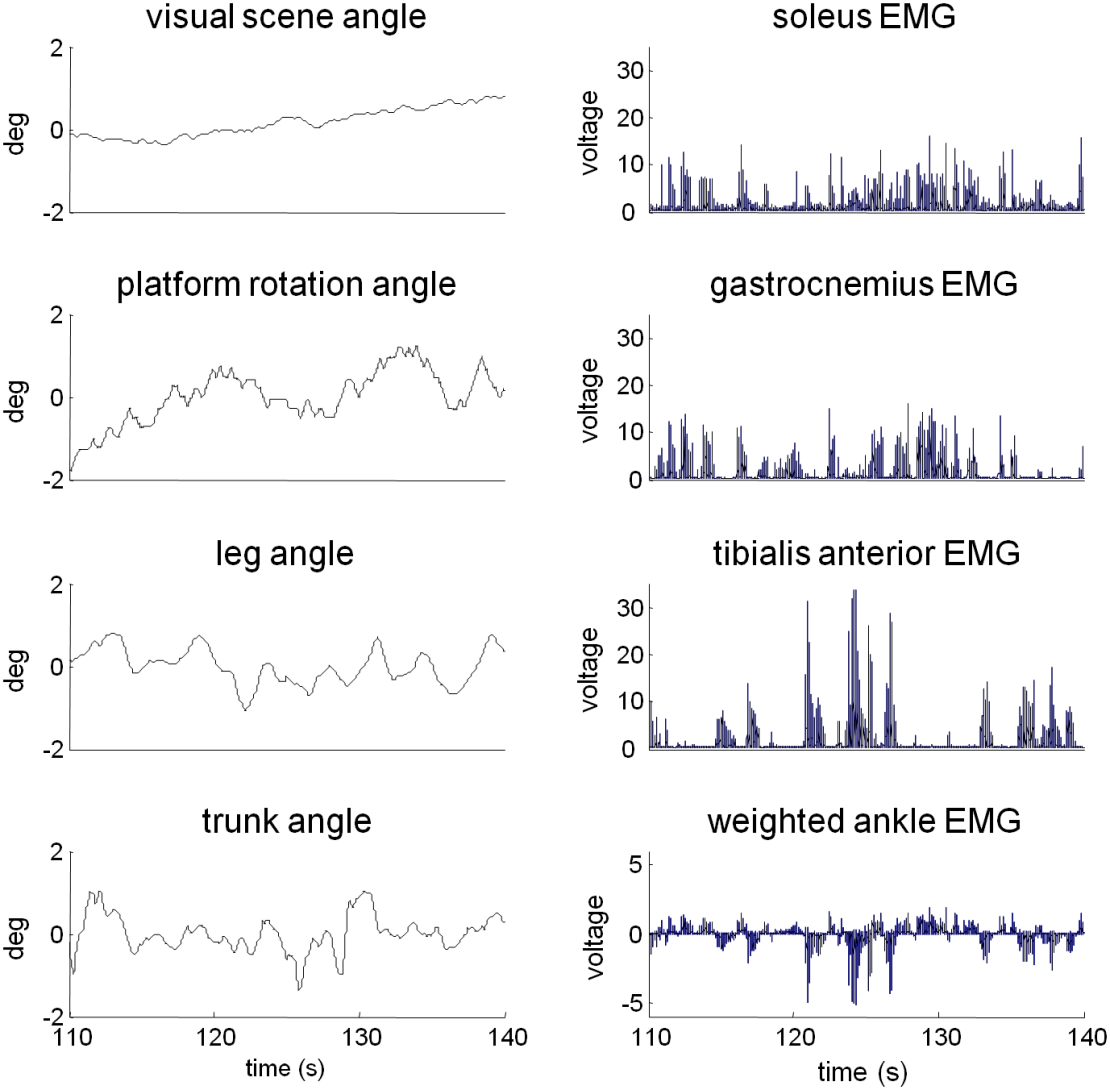
Perturbation, kinematics and ankle EMG of a single trial of a single subject in the high amplitude platform: low amplitude vision condition. Mean values were subtracted from the visual scene, platform, trunk and leg angles. Individual EMGs were normalized by the root-mean-square value. The weighted ankle EMG for this subject had weights of.28 for the soleus,.23 for gastrocnemius, and -.48 for tibialis anterior. These weights maximized coherence to average of visual and platform signals (see *methods*).

Exemplar time series of trunk/leg segment angles and visual/platform stimuli in the high amplitude platform and low amplitude vision condition are presented in Figure 2. EMG signals of soleus, gastrocnemius and tibialis anterior show the typical anti-phase firing patterns observed between anterior and posterior muscles. These muscles contribute to the weighted ankle signal whose time series indicates coupling to the platform stimulus during this condition.

### Weighting of the Musculature

Across all frequencies, average coherence of weighted ankle EMG and weighted hip EMG with each stimulus was larger than individual muscles contributing to these weighted signals. Additionally, the weighted all-muscle signal showed higher coherence to each stimulus than either weighted ankle or weighted hip signals at all frequencies. Partial coherence between individual ankle muscles and the weighted ankle muscle signal adjusted for the all-weighted signal was quite low (.033) on average across all muscles and frequencies, and partial coherence between individual hip muscles and the weighted hip muscle signal was similarly low (.031) on average. These partial coherences indicate that grouping muscles by either ankle or hip does not provide a unique relationship between EMG and a weighted signal above that observed when considering both segments together in one signal. Due to the stronger linear relationship of weighted all-muscle signal compared to weighted ankle and weighted hip muscle signals, recorded EMG signals are considered to be scaled versions of a single input control signal [16] and we present FRFs for the weighted all-muscle signal. Coherence of the weighted all-muscle signal with vision was.23 on average across conditions and frequencies while coherence of the weighted all-muscle signal with the platform was.62 on average across conditions and frequencies.

The mean±SD of these weights averaged across subjects were .18±.11(0) for the soleus, .25±.17(0) for the gastrocnemius, -.14±.10(2) for the tibialis anterior, .17±.18(3) for the biceps femoris, -.14±.17(3) for the rectus femoris, -.06±.08(4) for the rectus abdominus and .06±.08(4) for the erector spinae. Our weighting method did allow weights to be 0, yet this was quite infrequent and the number of subjects for which this occurred is noted in parentheses.

### Frequency Response Functions

**Figure 3:**
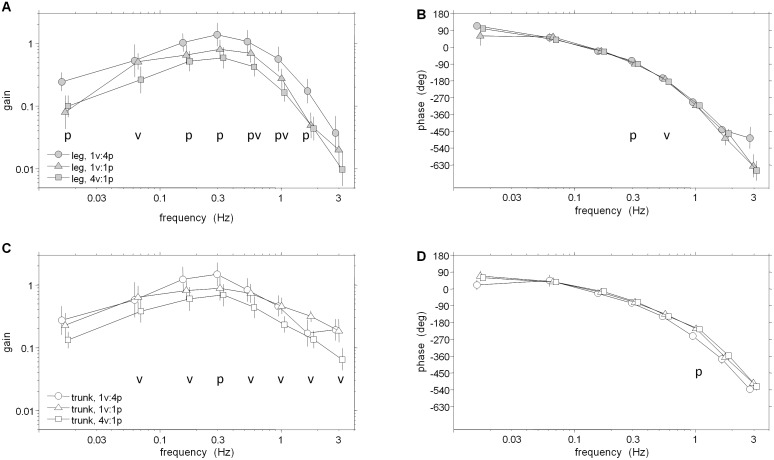
FRFS from visual scene angle to segment angles. A–B: Gain and phase of FRF from visual scene angle to leg segment angle. C–D: Gain and phase of FRF from visual scene angle to trunk segment angle. Error bars indicate bootstrapped standard error. Symbols p and v at individual frequency bins indicate a significant effect of increasing the amplitude of the visual perturbation or platform perturbation, respectively.

**Figure 4:**
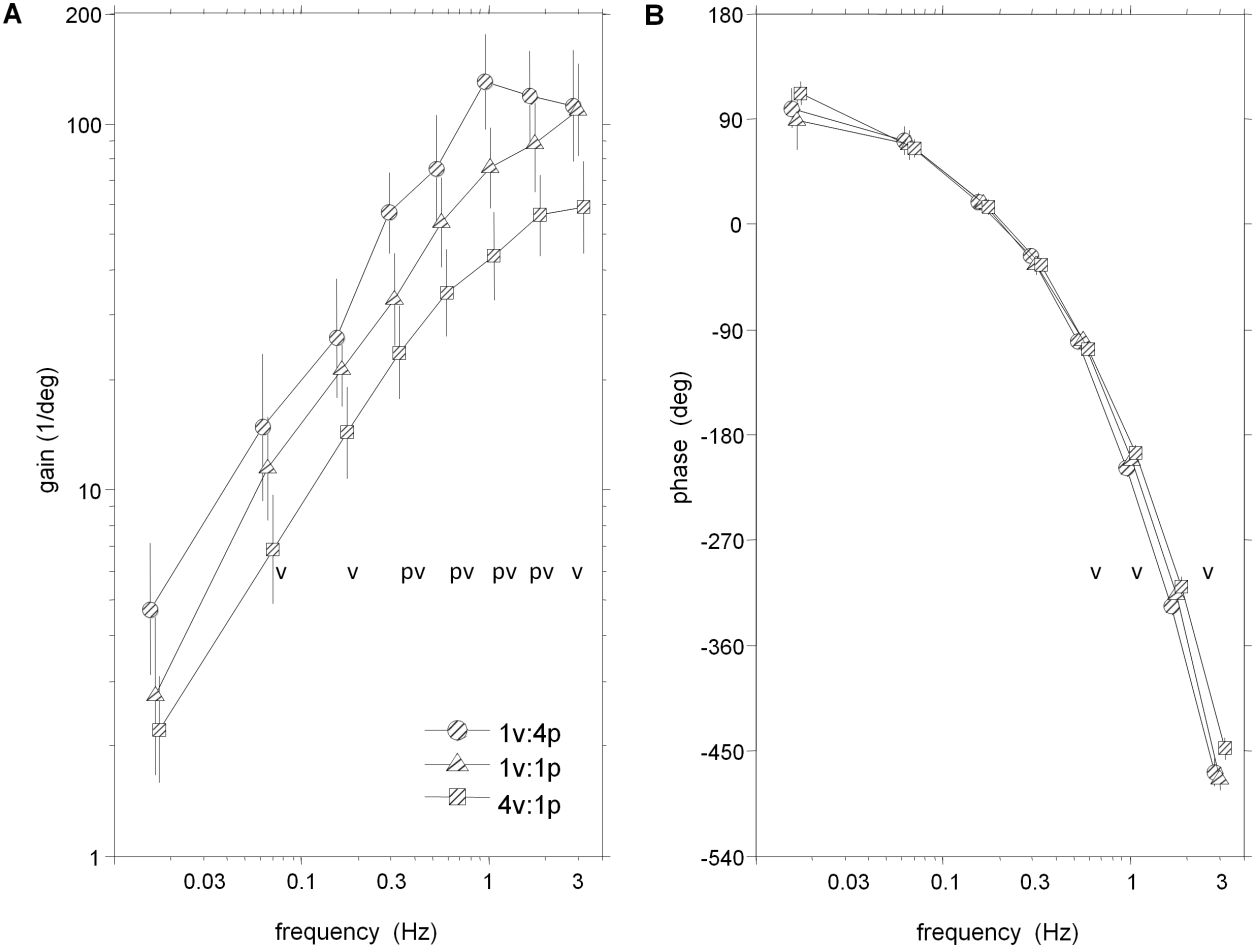
FRF from visual scene angle to weighted EMG. A: Gain of weighted EMG (all seven muscles from both ankle and hip) from visual scene angle. B: Phase of weighted EMG from visual scene angle. Error bars indicate bootstrapped standard error. Symbols p and v at individual frequency bins indicate a significant effect of increasing the amplitude of the visual perturbation or platform perturbation, respectively.

**Figure 5:**
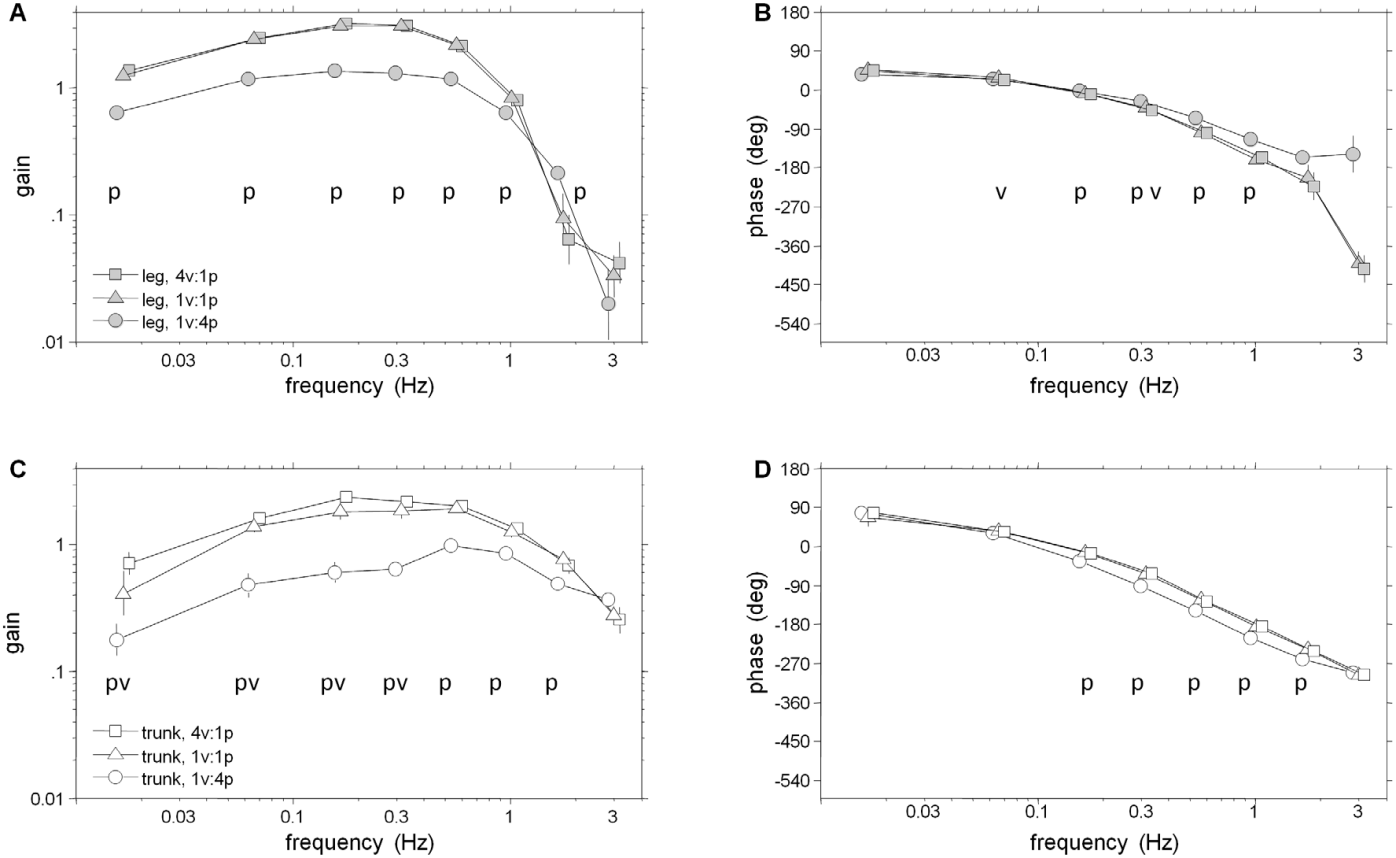
FRFS from platform angle to segment angles. A–B: Gain and phase of FRF from platform angle to leg segment angle. C–D: Gain and phase of FRF from platform angle to trunk segment angle. Error bars indicate bootstrapped standard error. Symbols p and v at individual frequency bins indicate a significant effect of increasing the amplitude of the visual perturbation or platform perturbation, respectively.

**Figure 6:**
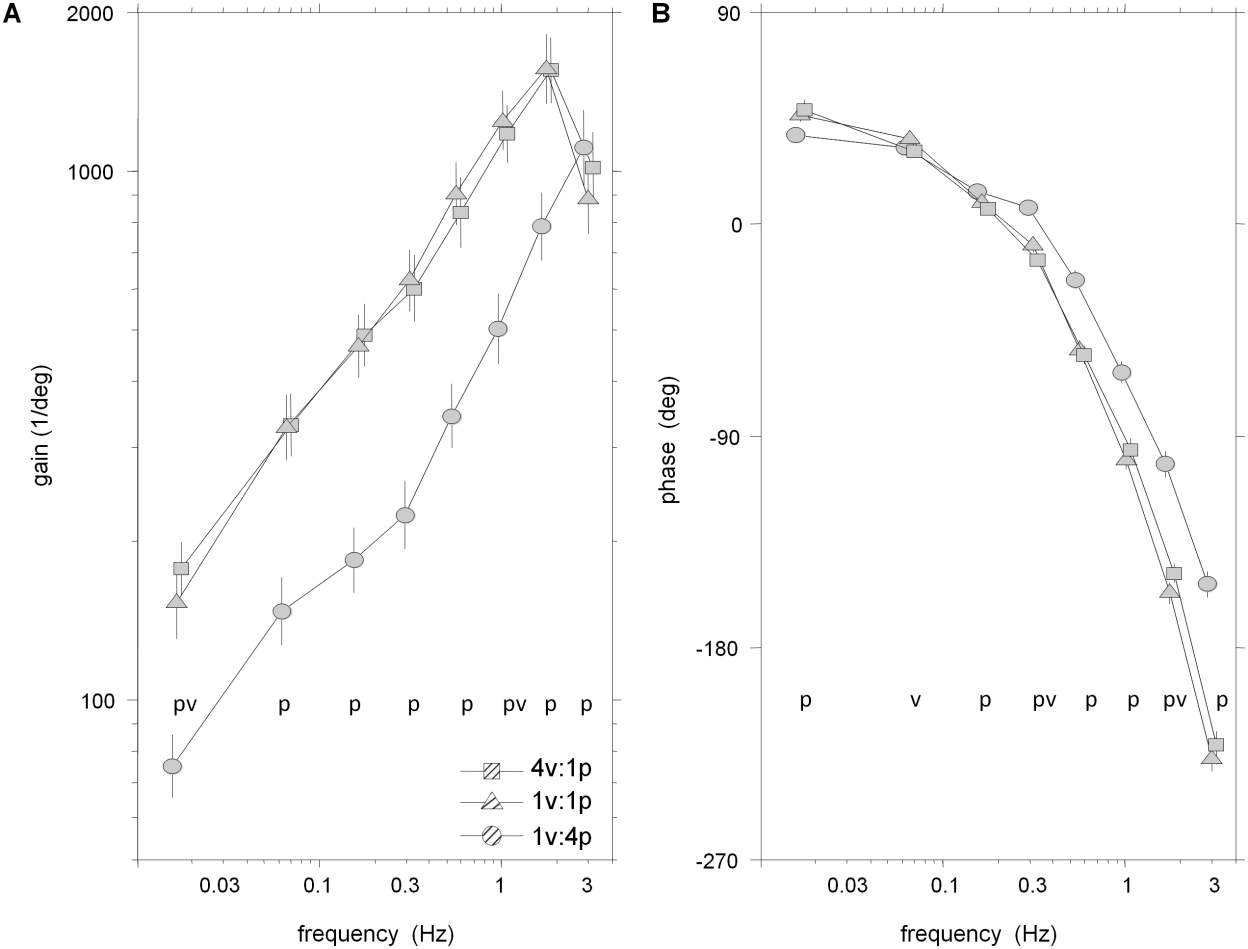
FRF from platform angle to weighted EMG. A: Gain of weighted EMG (all seven muscles from both ankle and hip) from platform angle. B: Phase of weighted EMG from platform angle. Error bars indicate bootstrapped standard error. Symbols p and v at individual frequency bins indicate a significant effect of increasing the amplitude of the visual perturbation or platform perturbation, respectively.

Frequency response functions (FRFs) of segment angles or muscular activity relative to the visual/platform stimuli in Figures 3–6 reveal clear changes in gain as a function of condition. When gain to a particular stimulus changes due to a change in that stimulus amplitude across conditions, gain modulation is interpreted as intra-modality reweighting. Inter-modality reweighting is observed when gain relative to a constant stimulus changes because of a change in amplitude of the other stimulus (e.g., visual gain changes due to change in platform amplitude).

#### Kinematic and muscular reweighting to visual drive

Figure 3 shows FRFs from visual scene angle to segment angles. Averaged across the eight frequency bins, log-gain for the leg segment was highest in the 1v∶4p condition, the condition in which visual information about self-motion was most reliable and ankle proprioceptive information about self-motion was least reliable. Log-gain was significantly smaller in the 1v∶1p condition (*p* = 0.006), indicating inter-modal down-weighting of visual information when proprioceptive information becomes more reliable. There was also a non-significant decrease from the 1v∶1p condition to the 4v∶1p condition (*p* = 0.052). In the trunk, a decrease in log-gain from the 1v∶1p condition to the 4v∶1p condition (p<.001) was observed, indicating an intra-modal down-weighting of visual information when it becomes less reliable. Inter-modal reweighting for the trunk across frequencies was not significant (*p* = 0.67). As observed in Figure 3A/C, however, all reweighting effects were detected when testing individual frequency bins: inter-modal reweighting was significant in bins 1 and 3–7 for the legs and bin 4 for the trunk (p<0.05); intra-modal reweighting was significant in bins 2 and 5–6 for the legs and bins 2–3 and 5–8 for the trunk.

Phase for the leg and trunk segments, shown in Figures 3B and 3D, monotonically decreased from +90 deg at low frequencies to approximately −540 deg at the highest frequencies in all conditions. Phase main condition effects were not significant for the legs (*p* = 0.24) and were significant for the trunk (*p* = 0.03) as a phase lag was observed for the trunk in the 1v∶4p condition compared to the 1v∶1p condition.

Unlike kinematic responses, EMG gains relative to the visual scene showed an increasing gain pattern across all frequencies (Fig. 4). However, reweighting was similar for EMG and kinematic responses. Averaged across the eight frequency bins, there was no detectable difference in the reweighting pattern of the leg, trunk and EMG response variables, as indicated by a non-significant Condition×Variable interaction for log-gain (*p* = 0.12). For the EMG response, log-gain was highest in the 1v∶4p condition, smaller in the 1v∶1p condition (inter-modal reweighting; *p* = 0.002) and decreased further in the 4v∶1p condition (intra-modal reweighting; *p*<.001). For individual frequency bins, inter-modal reweighting was significant for bins 4–7 and intra-model reweighting was significant for bins 2–8 (p<.05).

Phase between muscle activity and vision was approximately +90 deg at low frequencies and monotonically decreased as stimulus frequency increased (Fig. 4B). At all frequency bins, there were no significant phase differences between the low amplitude visual conditions. Interestingly, those EMG FRFs in the low amplitude platform conditions are in-phase up to bin 4 where the EMG response to 4v∶1p leads in the majority of remaining frequency bins (p<.05).

#### Kinematic and muscular reweighting to platform drive

FRFs of trunk/leg segment angles relative to platform angle shown in Figure 5 reveal intra-modal reweighting in both segments and inter-modal reweighting solely in the trunk segment. Averaged across the eight frequency bins, significantly smaller log-gain of the legs (p = .005) and trunk (p<.001) in the 1v∶4p condition compared to the 1v∶1p condition demonstrates intra-modal reweighting of the platform stimulus. Similar log-gain (p = .924) in the 1v∶1p and 4v∶1p conditions show a lack of inter-modality reweighting in the leg, as an increase in visual stimulus amplitude did not change platform gain. In the trunk, however, significantly (p = .02) larger log-gain to platform in the 4v∶1p compared to the 1v∶1p condition occurred, indicating inter-modal reweighting of the platform stimulus. As observed in Figure 5A/C, these reweighting effects were also detected when testing individual frequency bins: intra-modal reweighting was significant in bins 1–6 for the legs and bins 1–7 for the trunk (p<0.05); inter-modal reweighting was significant in bins 1–4 for the trunk. There was also a “cross-over” in gains of the leg as gain to the platform stimulus in the 1v∶4p condition was larger than that observed in the 1v∶1p condition in the seventh bin. This cross-over also occurred in the trunk in the eigth bin, yet it was non-significant.

Leg and trunk phase in low amplitude platform conditions were not different in the majority of frequency bins (p<.05) when testing individual frequency bins. Phase of the leg in the 1v∶4p condition significantly led the 1v∶1p condition at bins 3–6 (Fig. 5B). In a similar frequency range (bins 3–7), however, phase of the trunk in the 1v∶1p condition significantly led the 1v∶4p condition (Fig. 5D). Phase main condition effects were not significant for the legs (*p* = 0.281) and were for the trunk (*p* = 0.012) as a significant phase lag was observed for the trunk in the 1v∶4p condition compared to the 1v∶1p condition.

Similar to the leg segment angle, gain of EMG relative to platform rotation in Figure 6A indicates intra-modal reweighting with little evidence for inter-modal reweighting. Averaged across the eight frequency bins, a significant Condition×Variable interaction for log-gain (*p* = 0.03) to platform stimuli was observed, but no differences were found between EMG and the leg segment for the magnitude of log-gain changes due to changing conditions. The significant Condition×Variable interaction to the platform motion stimulus is due to the inter-modal reweighting observed in the trunk segment to the platform stimulus that is not observed in either the leg segment or EMG.

Averaged across the eight frequency bins, significantly (p<.001) smaller log-gain of EMG relative to the platform were observed in the the 1v∶4p condition compared to the 1v∶1p condition, indicating intra-modal reweighting of muscular activity. Significant differences between the low amplitude platform conditions were not observed (p = .45), supporting a lack of inter-modal reweighting to the platform stimulus. When testing at individual frequency bins, significantly higher gains in the 1v∶1p condition compared to the 1v∶4p conditions were found at almost all frequencies. Only at the highest frequency did this pattern change abruptly, with higher gain in the 1v∶4p condition in bin 8. Inter-modal reweighting of the platform stimulus was suggested in few instances. EMG gains were lower in the 1v∶1p condition than the 4v∶1p condition (p<.05) in only two frequency bins (1, 6).

Seen in Figure 6B, phase of EMG relative to platform rotation was not different in the majority of frequency bins (1,3,5,6,8) between low amplitude platform conditions. The phase relationship between the intra-modal conditions, however, reveals higher phases in the 1v∶4p condition than the 1v∶1p condition in bins 3–8 (p<.05). This relationship is also significant (p<.001) when phases are averaged across frequencies.

## Discussion

Although many studies have systematically investigated the interaction of visual and support surface inputs during postural control [5], [10]–[11], [24]–[25], this study was the first designed to investigate the presence of inverse-gain reweighting [6] as a rule for sensor fusion between these two sensory modalities. Since rotations of the visual scene and support surface were uncorrelated and simultaneously presented throughout conditions, gains to both perturbations could be independently measured, allowing us to investigate both intra- and inter-model sensory reweighting. Increasing the amplitude of support-surface rotations produced inverse-gain reweighting at low frequencies for both kinematic and EMG responses, that is, decreased gain to the support-surface rotation (intra-modal reweighting) and increased gain to the visual-scene rotation (inter-modal reweighting). However, increasing the amplitude of the visual-scene rotations did not produce significant changes in the gain to support-surface rotations in the majority of response variables, only decreased gain to the visual-scene rotations. These findings support the notion that the sensory integration scheme between these two sensory modalities is asymmetric, favoring the influence of somatosensory input at the surface of support during standing postural control.

### Responses to Visual-scene Movement have all the Traits of Sensory Reweighting

In the Introduction we hypothesized that changes in gain across conditions with different perturbation amplitudes reflect sensory reweighting and therefore should exhibit three key properties∶ (i) decreases in gain when the perturbation of the given sensory modality increases (intra-modal reweighting); (ii) increases in gain when the perturbation of a different sensory modality increases (inter-modal reweighting); (iii) equivalent percentage changes in EMG, leg and trunk gains. In our study, gain to visual-scene movement exhibited all three properties of sensory reweighting. When the amplitude of visual-scene movement increased, gain to the visual-scene movement decreased, demonstrating the intra-modal property. When the amplitude of platform movement increased, gain to the visual-scene movement increased, demonstrating the inter-model property. Both intra- and inter-modal changes in log-gain were not significantly different for the EMG, leg and trunk responses, consistent with equivalent percentage differences for all three responses.

Finally, we note that sway-referencing the platform in previous studies [5], [10] and increasing the amplitude of platform movement in the present study both increase gains to visual-scene movement. Both effects are consistent with inter-modal reweighting since both changes in platform movement make somatosensation (i.e., ankle proprioception, foot tactile sensation) a less reliable indicator of self-motion.

### Responses to Platform Movement Exhibit Mixed Effects

To test the hypothesis that changes in visual-scene movement produce inverse gain reweighting, FRFs to the platform movement were also computed. When the amplitude of the visual-scene motion was increased and the platform amplitude was kept the same, both EMG and leg segment angle gains to the platform motion did not show any significant changes. This is likely because our change in visual-scene motion was not large enough to detect the inter-modal effect on gains to platform motion. Peterka (2002) found that the extreme case of changing the visual scene from fixed to sway-referenced did increase the kinematic gain to platform motion [5]. However, this change in gain was not as large as the change in gain to visual-scene motion when the platform went from fixed to sway-referenced. Using a feedback postural control model, Peterka used these kinematic responses to estimate that under normal conditions proprioception provides most of the sensory information used to estimate self-motion. This may lead to a ceiling effect in which the inter-modal effect of vision on proprioception is necessarily less than the inter-modal effect of proprioception on vision. It is also possible that increasing platform motion led to an increase in use of vestibular sensory input. Inter-modal reweighting of vestibular input has been observed previously in the form of increased responses to the same galvanic vestibular stimulus (GVS) when the amplitude of support surface motion increased [11]. Recently, a sensory reweighting investigation simultaneously perturbing three modalities (vision, vestibular, somatosensation) showed that the specific response to the same GVS increased when either increasing amplitude of visual scene motion or turning on vibration at the Achilles tendon [26]. Relative contributions of either change in modality to the change in GVS response were not extracted in that study, further supporting the need for more experimental studies on how weighting of the three primary sensory modalities interact simultaneously to ensure upright stance.

Responses to platform movement did show effects consistent with intra-modal reweighting in EMG and both segment angles measured. When platform amplitude was increased while keeping visual amplitude the same, gains of the leg and trunk segments to the platform movement decreased. This intra-modal effect can be interpreted as downweighting of somatosensory information and has been previously observed in platform-induced postural sway [5], [27]. However, this intra-modal effect was only observed at low frequencies. At higher frequencies, a “cross-over” occurred in both segments where the gain to this high-amplitude platform motion is stronger than the gain to the low-amplitude platform motion (Fig. 5A/C). In EMG responses, a strong intra-modal effect is also observed in the low-mid range of frequencies while there is also a cross-over effect at higher frequencies (Fig. 6A).

To interpret these results, one must take into account that platform motion can produce both active and passive responses. That is, subjects respond actively to a somatosensory perturbation while platform motion can also physically perturb subjects to initiate a passive response of the body to the mechanical perturbation. Active responses depend on sensory feedback mediated by the nervous system and occur after some feedback time delay, whereas passive responses are due to the viscoelastic properties of muscle and tendon and occur without any delay. In addition to their dependence on frequency, active responses to support-surface perturbations also depend on perturbation amplitude due to nonlinearities such as sensory re-weighting (e.g., [5]). Although passive responses are usually modeled as being linear (e.g., [5], [13]), there is evidence of nonlinear passive musculotendon properties [28], so that passive responses may also depend on both the frequency and amplitude of support-surface perturbations. One additional consideration is that in models with low-pass filtering from muscle activation to joint torque (e.g., [29]), passive responses dominate the total response at high frequencies. Taken together, these factors suggest the possibility that the cross-over effect observed at high frequencies may be due to the increasing dominance of nonlinear passive responses. Additional experimental and modeling studies would be necessary to test this possibility.

In a similar study [5], both smaller phase lags of the COM to increasing amplitude of support-surface rotations and the “cross-over” in gains seen here were observed. In our study, which considers a two-segment body, smaller phase lags were observed in responses of leg and all-muscle weighted EMG while larger phase lags were observed in the trunk in similar frequency ranges. Gains, however, do converge in the vicinity of 2 Hz as Peterka (2002) has found for FRFs to support surface rotations up to 2.48 Hz [5]. Interestingly, Peterka (2002) also observed the cross-over effect at higher perturbation frequencies in a freestanding condition and not in a condition where subjects’ movements were constrained to rotation only at the ankles via a backboard. This suggests that higher frequencies are where the effects of increasing the platform amplitude on the passive multi-segment body dynamics are observed.

Further study is warranted to understand how passive and active postural control mechanisms contribute to responses to support-surface perturbations at higher frequencies. It is clear from this study, however, that somatosensory input at the surface of support has a large, asymmetric influence on the sensory integration scheme at a wide range of frequencies for standing postural control.
